# Capillary electrophoresis for the characterization of quantum dots after non-selective or selective bioconjugation with antibodies for immunoassay

**DOI:** 10.1186/1477-3155-6-10

**Published:** 2008-10-01

**Authors:** Mark Pereira, Edward PC Lai

**Affiliations:** 1 Department of Chemistry, Ottawa-Carleton Chemistry Institute, Carleton University, Ottawa, ON K1S 5B6, Canada

## Abstract

Capillary electrophoresis coupled with laser-induced fluorescence was used for the characterization of quantum dots and their conjugates to biological molecules. The CE-LIF was laboratory-built and capable of injection (hydrodynamic and electrokinetic) from sample volumes as low as 4 μL via the use of a modified micro-fluidic chip platform. Commercially available quantum dots were bioconjugated to proteins and immunoglobulins through the use of established techniques (non-selective and selective). Non-selective techniques involved the use of EDCHCl/sulfo-NHS for the conjugation of BSA and myoglobin to carboxylic acid-functionalized quantum dots. Selective techniques involved 1) the use of heterobifunctional crosslinker, sulfo-SMCC, for the conjugation of partially reduced IgG to amine-functionalized quantum dots, and 2) the conjugation of periodate-oxidized IgGs to hydrazide-functionalized quantum dots. The migration times of these conjugates were determined in comparison to their non-conjugated QD relatives based upon their charge-to-size ratio values. The performance of capillary electrophoresis in characterizing immunoconjugates of quantum dot-labeled IgGs was also evaluated. Together, both QDs and CE-LIF can be applied as a sensitive technique for the detection of biological molecules. This work will contribute to the advancements in applying nanotechnology for molecular diagnosis in medical field.

## Background

Quantum dots (QDs) are fluorescent nanoparticles that receive increasing recognition as a viable alternative (to conventional organic fluorophores) for molecular labeling. Their quantum mechanical and electronic characteristics give QDs unique optical properties that are advantageous in the fields of bioanalytical, biomedical and biophotonic research. Such optical properties include size-tunable emission wavelengths, broad excitation wavelengths, long fluorescence lifetimes, large Stokes shifts, and high quantum yields [1-3]. Other advantageous properties include resistance to photo- and chemical- degradation and their capability for performing multiplexing experiments [3]. QDs are relatively large particles, with typical diameters ranging from 1–10 nm [1]. The inorganic core (typically a semiconductor) is responsible for their fluorescent properties. This core is typically surrounded by a shell (ZnS is common) for protection from chemical- and photo-oxidation [2]. The shell also provides a means of functionalizing the QD with carboxylic acids or primary amines, for good solubility in aqueous solutions and relative ease of specific labeling reactions [1].

**Figure 1:**
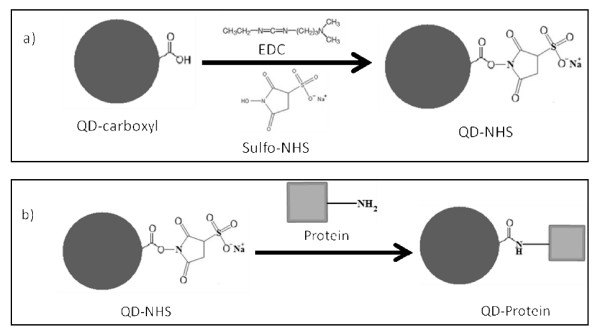
**Non-selective bioconjugation reaction scheme of carboxylated QDs (QD-COOH) to amine-containing proteins**. This two-step reaction involves a) the activation of QD-COOH with EDC/sulfo-NHS, resulting in a semi-stable active ester (QD-NHS), and b) the nucleophilic reaction between the QD-NHS and amine-containing protein, forming a QD-protein conjugate via a stable amide bond.

QDs, often applied for the labeling of biological molecules (proteins, peptides, antibodies, etc.), require specific techniques for their conjugation [4-7]. The most popular bioconjugation technique involves the use of a zero-length crosslinker, 1-ethyl-3- [3-dimethylaminopropyl]carbodiimide hydrochloride (EDCHCl) [1-4,6,7], in the presence of a hydrophilic active group, N-hydroxysulfosuccinimide (sulfo-NHS) [8], for the formation of a stable amide bond between carboxylic acid-functionalized QDs (QD-COOH) and any biomolecules containing a primary amine [9] (Figure 1).

This method, while proven to yield exclusively QD-protein conjugates in a controlled manner, randomizes the location on a protein to which conjugation can occur, resulting in a non-selective bioconjugation [9]. Despite high bioconjugation efficiencies, this can be detrimental in the case where an immunoassay is to be performed next. For instance, a labeled protein serving as an antigen might lose its antigenicity (ability to bind an antibody) when conjugated to a large QD. A similar concern can be conveyed if an antibody were conjugated in a region close to the antigen-binding site (the hypervariable region). Either one of these variations can significantly reduce the efficiency of immunoassay applications [9].

**Figure 2:**
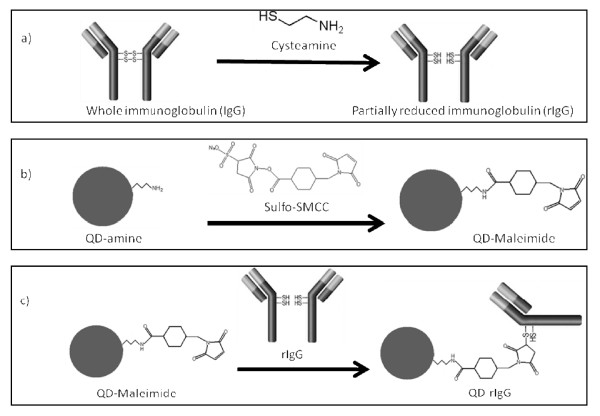
**Selective bioconjugation reaction scheme of amino QDs (QD-amine) to free sulhydryl-containing IgG antibodies**. The reaction involves a) the mild reduction of IgG with cysteamine to yield partially reduced IgG antibody fragments (rIgG); b) the activation of QD-NH_2 _by nucleophilic reaction with NHS-moiety of sulfo-SMCC, resulting in maleimide-functionalized quantum dot (QD-maleimide); and c) the rIgG and QD-maleimide conjugation (QD-rIgG) via the formation of a thioether bond.

Other techniques make effective use of selective bioconjugation, targeting specific sites on the protein. These include the use of a heterobifunctional crosslinker such as sulfosuccinimidyl-4-(*N*-maleimidomethyl)cyclohexane-1-carboxylate (sulfo-SMCC) [9-11]. In the case for antibodies, as shown in Figure 2 below, sulfo-SMCC can form stable amide bonds to amine-functionalized QDs (QD-NH_2_) [9]. The resultant QDs, through sulfo-SMCC's maleimide region, can next form stable a thioether bond with a sulfhydryl-exposed antibody [9]. Mild reducing reagents such as cysteamineHCl (or DTT) can selectively cleave the disulfide bonds (hinge region) connecting the IgG heavy chains, while leaving the other disulfide bonds that make up the antigen binding site (hypervariable region) unaffected, thus producing a partially reduced IgG (rIgG) [12]. In addition, the resulting exposed sulfhydryls (hinge region) are sufficiently far away (from the hypervariable region) for QD-bioconjugation to occur. The resulting quantum dot-conjugated half antibody (QD-rIgG) will allow an immunoreaction to proceed readily.

**Figure 3:**
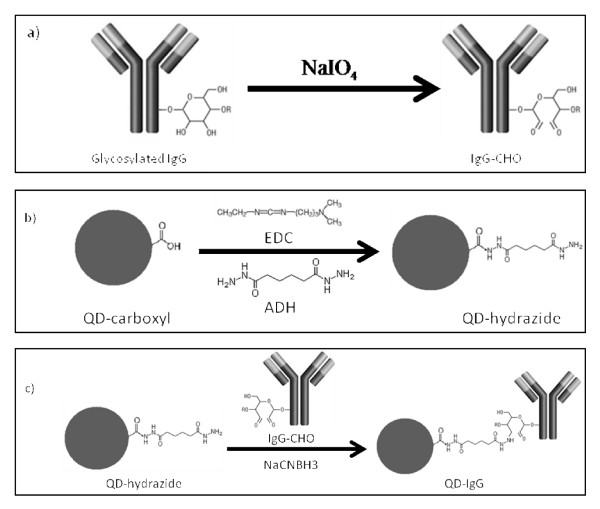
**Selective bioconjugation reaction scheme of hydrazide QDs (QD-hydrazide) to aldehyde-containing IgG antibodies (IgG-CHO)**. The reaction involves a) mild periodate oxidation of glycosylated IgG, yielding IgG-CHO; b) synthesis of QD-hydrazide via derivatization of QD-COOH with EDC/ADH; and c) conjugation of QD-hydrazide with IgG-CHO via formation of hydrazone linkage to yield QD-IgG.

Reductive amination is a bioconjugation technique popular in the labeling of glycoproteins. Taking advantage of the polysaccharide chains within the Fc region of an antibody, it can allow bioconjugation to occur relatively far away from the antigen binding site. Through oxidation (using sodium periodate) of the carbohydrate hydroxyls, the aldehydes formed are highly reactive toward primary amines and hydrazides [9]. This makes QD-NH_2 _or QD-COOH (derivatized with adipic acid dihydrazide (ADH)) suitable candidates for conjugation [9]. In addition, selective bioconjugation can occur without a proceeding reduction reaction, thus retaining the integrity of the antibody (Figure 3).

Capillary electrophoresis (CE) has seen increasing use in the separation and characterization of inorganic nanoparticles (Ag, Au, TiO_2_, Al_2_O_3_, Fe_2_O_3_) [13-17], polystyrene microspheres [18], biomolecules (proteins, peptides) [19-30], QDs [31], QD-conjugates with bovine serum albumin (BSA) and horse radish peroxidase (HRP) [7], and QD-conjugates with *Ulex europaeus *(UEA-1) and anti-von Willebrand factor (anti-vWF) [32]. CE has also been used for immunoassays involving hepatitis B, prion protein, alpha-fetoprotein, etc [24-30]. Recently, a CE-based immunoassay involving QDs conjugated to anti-IgM antibodies followed by immuno-conjugation to its complimentary antigen IgG was performed with satisfactory results [33]. Another recent advancement involved the CE-characterization of QDs (of differing emission wavelengths) exclusively conjugated to biotin and streptavidin [34]. Their work followed the characterization of the conjugates' affinity to each other via strong biotin-streptavidin interactions. However, present publications reporting the use of QDs in CE-based immunoassays are very preliminary, due in part to a QD-biomolecule conjugate's (and immunoconjuagte's) complex charge-to-size ratio. Thus, more research is required in its development as a fast and efficient method for performing immunoassays.

In this paper, we report more preliminary results of covalently bioconjugating QDs to various biomolecules (proteins and immunoglobulins). These QD-conjugated biomolecules are characterized via a laboratory-built capillary electrophoresis instrument with laser-induced fluorescence detection (CE-LIF) [35]. The instrumental capabilities (comparable to commercial CE-LIF systems) include the use of a micro-sample injection platform that can load sample volumes as low as 4 μL [35]. We also discuss some of the challenges faced when performing bioconjugation through the various schemes described above. The purpose is to validate a fast, selective, and reproducible CE-LIF analysis method that can be efficient and robust. This work will evolve to perform QD-based immunoassays using CE-LIF as an effective separation and sensitive detection technique. The aim is to apply this research in the area of infectious biological materials that are generally present in relatively low concentrations and small volumes.

## Methods

### Chemicals and reagents

Boric acid (certified A.C.S.), sodium meta-periodate (crystalline, A.C.S. grade), sodium hydroxide (reagent grade) were purchased from Fisher Scientific (Ottawa, Ontario, Canada). CdSe/ZnS carboxy-terminated QDs (Maple Red-Orange, 620 nm) and CdSe/ZnS amine-terminated QDs (Maple Red-Orange, 620 nm) were purchased from Evident Technologies (Troy, NY, USA). EDCHCl, Sulfo-NHS, lysozyme (Lys), and MES buffered saline packs were purchased from Pierce Biotechnology. Sodium acetate (reagent grade) and hydroxylamine hydrochloride (reagent grade) was purchased from Anachemia. EDTA (0.1 M volumetric standard), ADH (= 98%), sulfo-SMCC (= 98%), DL-DTT (1 M in water solution), anti-human albumin (polyclonal IgG produced in rabbit), human serum albumin (HSA), cysteamine hydrochloride (Purum = 97.0%), 2-mercaptoethanol (14 M), 10× PBS concentrate, bovine serum albumin (BSA), horse myoglobin (Myo) cytochrome c (CytC), ethanolamine, and sodium cyanoborohydride (5 M in 1 M sodium hydroxide) were purchased from Sigma Aldrich. Coumarin 521 was purchased from Exciton (Dayton, Ohio, USA). Micro-centrifuge tubes (50 kDa and 100 kDa MWCO) were purchased from Fisher Scientific.

### Preparation of buffer solutions and stock solutions

All buffer solutions were prepared and pH-adjusted using sodium hydroxide (10 M, 5 M, and 1 M) and hydrochloric acid (1 M and 0.5 M). All CE separation buffers were filtered through a 0.45 μm membrane filter (Pall Corporation, Ann Arbor, MI, USA).

Carboxy- and amine- terminated QDs were used from supply stock (11 μM) without any prior treatment.

Stock solutions of EDCHCl (20 mM) and sulfo-NHS (50 mM) were prepared by dissolution of dry reagents in 0.1 M MES (pH 5.2) buffered saline and used immediately after preparation. Stock solutions of 2-mercaptoethanol (1 M) and hydroxylamine hydrochloride (1 M) were prepared and stored at room temperature.

Stock solutions of cysteamineHCl (100 mM) were prepared by dissolution of dry reagent in 1× PBS (pH 7.2), 10 mM EDTA and used immediately after preparation. Stock solutions of DTT (100 mM) were prepared by dilution of a 1 M DTT stock solution and used within 3 days of preparation.

Stock solutions of NaIO_4 _(100 mM) were prepared by dissolution of dry reagents in 0.1 M sodium acetate (pH 5.5) buffered saline. Preparation and storage was performed in minimal lighting and used immediately after use. Sodium cyanoborohydride (5 M in 1 N NaOH) was used as prepared from supplier. Stock solution of ethanolamine (1 M) was prepared by dissolution of dry reagent in distilled deionized water (ddw) and pH adjusted to 9.6.

Stock solutions (1 mg/mL) of bovine serum albumin (BSA), myoglobin (Myo), cytochrome c (CytC), and lysozyme (Lys), were prepared in 1× PBS (pH 7.2). Human serum albumin (HSA) was prepared in ddw (11 mg/mL). Anti-human albumin IgG (4 mg/mL) was prepared in 1× PBS (pH 7.2).

### Non-specific bioconjugation of whole IgG using EDCHCl/sulfo-NHS

A mixture containing 2 mM EDCHCl, 5 mM sulfo-NHS, and 1.1 μM carboxy-terminated QDs (QD-carboxyl) was prepared in 0.1 M MES, pH 6.0 and incubated for 15 minutes at room temperature. The remaining unreacted EDC was quenched with the addition of 2-mercaptoethanol (1 M) to a final concentration of approximately 20 mM and the mixture was left to stand for 10 minutes. The activated QDs were purified of unreacted reagents and byproducts by dialysis using 100 kDa MWCO microcentrifuge tubes and re-suspended in 1× PBS (pH 7.2) containing dissolved protein. The reaction proceeded for 2 hours with gentle mixing. The reaction was quenched with addition of hydroxylamine hydrochloride (1 M) to a final concentration of approximately 10 mM. The bioconjugation mixture was left to stand for 10 minutes at room temperature prior to purification by dialysis using 100 kDa MWCO microcentrifuge tubes. The mixture was analyzed by CE-LIF and stored at 4°C.

### Selective bioconjugation of reduced IgG (rIgG) using cysteamineHCl or DTT and sulfo-SMCC

A mixture containing approximately 1 mg/mL rabbit anti-human albumin IgG and cysteamineHCl (concentration ranging from 0.1 mM to 100 mM) was incubated at 37°C for 90 minutes in 0.1 M sodium phosphate (pH 7.0), 0.15 M, 0.01 M EDTA. The resulting partially reduced antibody (rIgG) was purified of byproducts and unreacted compounds via dialysis using a 50 kDa MWCO microcentrifuge tube with successive washings of 0.1 M sodium phosphate (pH 6.8), 0.15 M NaCl, 0.01 M EDTA buffer. The rIgG was temporarily stored at 4°C until use for QD coupling.

Amine-functionalized QDs (QD-amine) were added to a 50 mM sodium phosphate (pH 7.2) solution containing sulfo-SMCC (8.8 mM) and incubated at room temperature for 60 minutes with gentle mixing. The maleimide-activated QDs (QD-maleimide) were purified of unreacted cross-linker via dialysis using 100 kDa MWCO microcentrifuge tubes at room temperature with successive washings of 0.1 M sodium phosphate (pH 6.8), 0.15 M NaCl, 0.01 M EDTA buffer. The purified QD-maleimide was used immediately.

The rIgG and QD-maleimide were combined and incubated overnight at 4°C. Purification of QD-rIgG of "free" rIgG in solution was performed via dialysis using 100 kDa MWCO microcentrifuge tubes. The purified QD-rIgG was washed several times with ddw. The purified QD-rIgG was analyzed by CE-LIF and stored at 4°C.

### Selective bioconjugation of whole IgG using EDC/ADH and sodium meta-periodate

A mixture containing 20 μL QD-carboxyl (11 μM), 16 mg EDCHCl, and 32 mg ADH were incubated in 1 mL 1× PBS for 4 hours at room temperature with gentle mixing. The hydrazide-functionalized QDs (QD-hydrazide) were purified from excess reagents via dialysis using a 100 kDa MWCO microcentrifuge tube. The purified concentrate was stored at 4°C until analysis by CE-LIF and IgG-CHO coupling.

A 500 μL mixture containing approximately 1 mg/mL rabbit anti-human albumin IgG and sodium meta periodate dissolved in 0.1 M sodium acetate buffered saline was incubated in the absence of light for 1 hour at room temperature with gentle mixing. The oxidized IgG (IgG-CHO) was purified of excess reagents via dialysis using a 100 kDa MWCO. The purified IgG-CHO was used immediately.

The IgG-CHO was combined with QD-hydrazide (50 μL total volume) and incubated overnight (14 hrs) at room temperature with gentle mixing. Stabilization of the hydrazone linkages were performed via the addition of 5 μL sodium cyanoborohydride (5 M in 1 N NaOH) with continued incubation for 1 hour. Unreacted aldehydes were blocked via addition of 25 μL of 1 M ethanolamine (pH 9.6) with continued incubation for 1 hour. Mixture was removed of excess sodium cyanoborohydride and ethanolamine via dialysis using 100 kDa MWCO. Mixture was not purified of unreacted IgG or QD.

### Immunoconjugation of QD-rIgG with corresponding antigen

A 10 μL aliquot of immunogen HSA (11 mg/mL) was added to a 300 μL solution of QD-rIgG (rabbit anti-human albumin) and incubated for 15 minutes at room temperature. The mixture was immediately analyzed be CE-LIF and later stored at 4°C.

### CE-LIF analysis

CE-LIF analysis of QDs, bioconjugates, and immunoconjugates were performed on a laboratory-built system described previously. A fused silica capillary (51 mm id, 362 mm o.d., *L*_t _= 58.5 cm, *L*_d _= 52.1 cm, and *L*_dw _= 2 mm) was flushed with 1.0 M NaOH, 0.1 M NaOH, DDW, and run buffer. Prior to each use, the capillary was equilibrated with the run buffer at an applied voltage of 25 kV for 10 min. Capillary temperature was maintained constant at 20.0°C by water from a PolyScience 1160A circulating bath (Niles, IL, USA). Hydrodynamic injections were performed by elevating the sample to 8 cm for 15 s. Microsample injections were performed using the sample port of a modified microfluidic chip as described previously [34]. An Extreme DPSS 473 nm, 500 mW solid-state diode laser (Seabrook, TX, USA) was used for fluorescence excitation. The LIF intensity was detected using a Hamamatsu model H7827-001 PMT (Bridgewater, NJ, USA) equipped with a 620 ± 5 nm interference filter. Spectral response of the PMT was 300–650 nm. The detector output signal was acquired through the Peak Simple Chromatography Data System.

## Results and discussion

### Use of EDCHCl/sulfo-NHS as a non-selective technique for bioconjugation of QDs to proteins

This non-selective technique for bioconjugation involved a two-step reaction using EDCHCl/sulfo-NHS to control the conjugate formation. Bioconjugation of proteins to carboxylated QDs have been performed with the use of EDC alone [7]. Despite the simplicity of a one-step reaction, the drawback involves a degree of uncontrollability during bioconjugation, forming unlabeled protein-protein conjugates and QD-protein polymers that can ultimately lead to precipitation. The use of sulfo-NHS was included to prevent these unwanted conjugate by-products and yield exclusively QD-protein conjugates. However, the number of proteins bound to a single QD may vary (depending on experimental conditions) and have yet to be determined.

**Figure 4:**
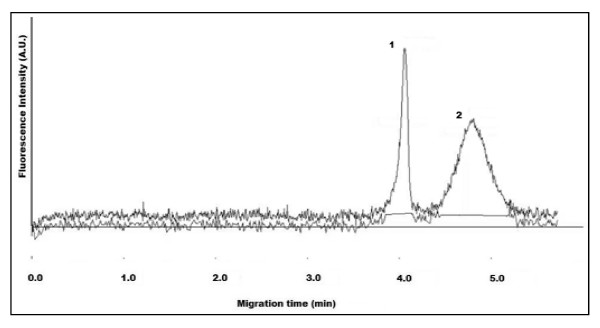
**Electropherogram of mixture containing QD-COOH (1) and BSA-conjugated QDs (QD-BSA) (2)**. CE buffer electrolyte used was 50 mM borate, pH 9.2. Gravity injection performed by elevating inlet capillary 7 cm for 5 s. Applied voltage for CE separation was 20 kV. Capillary temperature maintained at 20°C. Excitation source and detection wavelength was 473 nm and 620 nm, respectively.

Figure 4 illustrates the CE separation of carboxylated QDs (QD-COOH) (1) from their conjugation to BSA (QD-BSA) (2). The QD-BSA was detected at a longer migration time with respect to QD-COOH due to the inherent increase in the net negative charge of the conjugate. This was expected since the isoelectric point (pI) of BSA (~5.6) is much lower than the CE buffer pH (9.2) and thus expressing an increased number of negative charges that will ultimately influence the net-charge of the conjugate. The increase in peak width of the QD-BSA can be attributed to a number of factors, including the polydispersity of QDs during synthesis, the binding ratio of BSA to QDs, and the protein-capillary wall interactions that can take place with protein functionalized-QDs.

**Figure 5:**
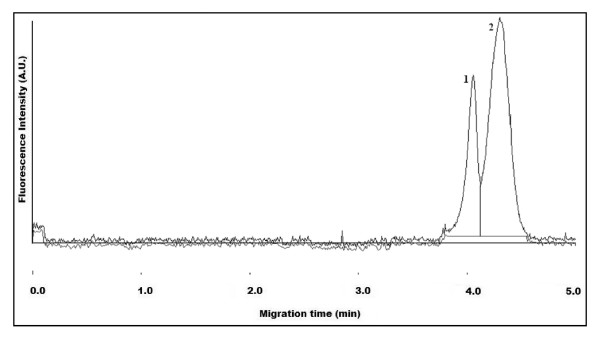
**Electropherogram of mixture containing QD-COOH (1) and myoglobin-conjugated QDs (QD-Myo) (2)**. CE buffer electrolyte used was 50 mM borate, pH 9.2. Gravity injection performed by elevating inlet capillary 7 cm for 5 s. Applied voltage for CE separation was 20 kV. Capillary temperature maintained at 20°C. Excitation source and detection wavelength was 473 nm and 620 nm, respectively.

Figure 5 illustrates the CE separation of QD-COOH (1) with their conjugation to myoglobin (QD-Myo) (2). The migration time of QD-Myo is also longer with respect to QD-COOH. However the differences are not substantial enough for baseline separation to occur. In comparison to QD-BSA, there may be a weakened net negative charge that is present on QD-Myo, since myoglobin has a pI value measured at ~7.2. In addition, there is a considerable size difference between BSA (MW~66 kDa) and Myo (MW~16.7 kDa) that may likely influence the respective conjugate's migration time. As both MW and pI can influence a protein's charge-to-size ratio, their conjugation to polydisperse QDs (each with possibly different binding ratios) will contribute to their respective migration times.

The chemistry of bioconjugating QD-COOH to proteins using EDCHCl/sulfo-NHS was attractive due to its versatility, as primary amines (lysine ε-amine and N-terminal α-amine) are present on many proteins. This ultimately led to the attempt of bioconjugating QD-COOH to proteins of increasingly higher pI, using cationic proteins such as cytochrome c and lysozyme. However, it was observed that the pI of proteins can play a determining factor in the efficiency of a bioconjugation. While the reaction was efficient in conjugating anionic proteins (BSA and myoglobin) to QD-COOH, it was unsuccessful in conjugating to cationic proteins (cytochrome c and lysozyme). It is suspected that the pI of cytochrome c (~10) and lysozyme (~11) maintained the primary amines (those accessible for conjugation) in a protonated state. This protonated state would render these proteins poor in a nucleophilic reaction with the NHS-activated QD-COOH (QD-NHS), thus inhibiting bioconjugation. The lack of a bioconjugation results in an eventual hydrolysis reaction with QD-NHS leading to the formation of the QD-COOH which can be identified using CE (data not shown).

Another drawback for the use of EDCHCl/sulfo-NHS for the formation of stable bioconjugates is the lack of specificity on the protein of interest. As numerous amine functional groups can be distributed throughout the surface of the protein, a bioconjugation involving such functional groups via a EDCHCl/sulfo-NHS reaction would lead to a randomization of crosslinking sites.

### Use of selective (heterobifunctional crosslinker) technique for bioconjugation of QDs to IgGs

**Figure 6:**
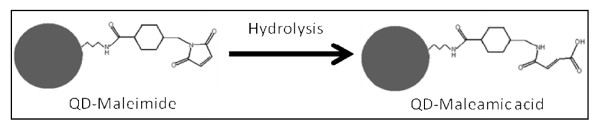
**Reaction scheme illustrating hydrolysis of sulfo-SMCC activated of QD-NH_2 _(QD-maleimide)**. Hydrolyzed QD-maleimide will contain maleamic acid moiety (QD-maleamic) unreactive towards free sulfhydryls.

The use of the heterobifunctional crosslinker sulfo-SMCC allowed for straightforward activation of amine-functionalized QDs (QD-NH_2_) via a nucleophilic reaction between the active ester on the crosslinker and the amine moiety of the QD. Despite the activated QD (QD-maleimide) being relatively stable at physiological pH, temperature is an important factor to control as higher temperatures (above room temperature) can accelerate hydrolysis reactions. Hydrolysis of the maleimide moiety will form maleamic acid that is unreactive towards free sulfhydryls (Figure 6). Characterization of the hydrolyzed QD-maleimide by CE detected a migration time similar to that for QD-COOH (data not shown).

**Figure 7:**
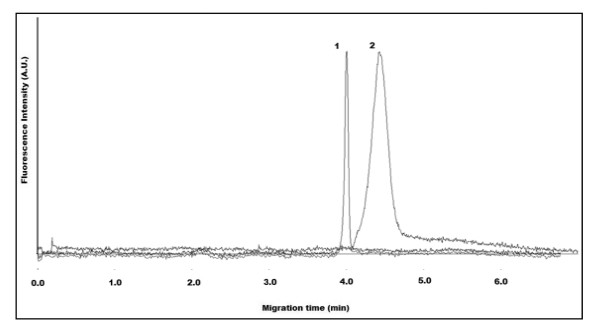
**Overlapping electropherograms illustrating QD-NH_2 _(1) and QDs conjugated to reduced antibodies QD-rIgG (2)**. IgG used for conjugation was rabbit anti-human albumin. CE buffer electrolyte used was 50 mM borate, pH 9.2. Gravity injection performed by elevating inlet capillary 7 cm for 5 s. Applied voltage for CE separation was 25 kV. Capillary temperature maintained at 20°C. Excitation source and detection wavelength was 473 nm and 620 nm, respectively

Due to the high-pH instability of QD-maleimide, CE characterization was not performed. However, it can be expected that the neutral charge present on the maleimide would compel the QD-maleimide to migrate more slowly, relative to the positively charged QD-amine prior to activation. The use of either cysteamineHCl (50–100 mM) or DTT (1–10 mM) as the reducing agent for IgGs provided similar results. However, both incubation time and temperature are dramatically different (90 min at 37°C for cysteamineHCl and 30 min at room temperature for DTT). Furthermore, the use of 50 kDa MWCO centrifuge filters allowed for retention of the partially-reduced IgG (rIgG), while removing unused reagents and byproducts. Combining of the QD-maleimide with rIgG at room temperature for at least 2 hours (or at 4°C overnight) provided similar results shown in Figure 7 below.

Figure 7 illustrates overlapping electropherograms of QD-NH_2 _(1) and their conjugation to the reduced anti-human albumin IgG (QD-rIgG) (2). The longer migration time observed for QD-rIgG can lead to the assumption that the rIgG exhibits a net negative charge in this CE separation buffer. Thus, when conjugated to the positively charged QD-NH_2_, the charge influence of the rIgG results in the conjugate displaying a smaller net positive charge. It is suspected that the IgG is comparable in acidity to the smaller proteins (BSA and myoglobin) used, however other factors including size and QD:biomolecule binding ratios need to be taken into consideration. Similar electropherograms were obtained when conjugating QD-NH_2 _to another IgG, anti-chicken lysozyme (data not shown). This can be attributed to IgGs having MWs typically at 150 kDa. However, IgG can range in pI from 6.4 to 9.0, due mainly to changes in their hypervariable region which can contain various charged residues. Thus, changes in CE separation buffer (particularly pH) could possibly influence the relative migration times of QDs conjugated to different IgGs and hence aid in selectivity and resolution. The observed migration time for the EOF was measured slightly earlier than the QD-NH_2 _(data not shown). This was unexpected since these observations would suggest QD-NH_2 _expressing a net negative charge. However, higher concentration borate buffers (greater than 200 mM) did measure the EOF at a later migration time than QD-NH_2 _(data not shown). The reason for the unexpected migration time for QD-NH_2 _at different borate concentrations may require further knowledge of the commercialized QD coating/functionalization process.

### Use of selective (hydrazone linkage) technique for conjugation of IgGs to QDs

**Figure 8:**
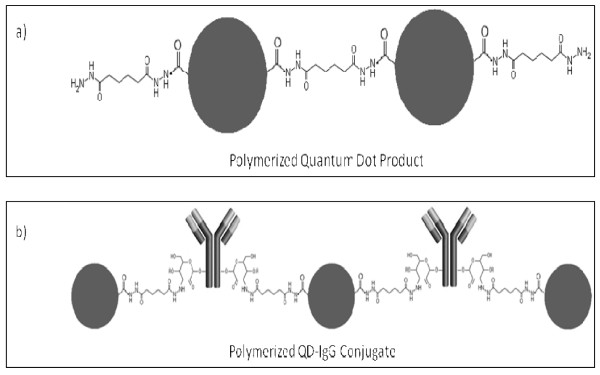
**Possible unfavorable polymer formation during following bioconjugation steps**. a) QD-hydrazide synthesis from QD-COOH, and b) QD-IgG bioconjugation from QD-hydrazide and IgG-CHO.

Conjugation of IgG-CHO with QD-NH_2 _is possible using reductive amination. However, the drawback is the degree of uncontrollability of the resulting conjugate, as undesirable IgG-IgG crosslinking can occur through the presence of primary amines on the IgGs surface. Thus, conjugating IgG-CHO with QDs functionalized with hydrazides was reasoned to be more selective as conjugation is occurring exclusively on the polysaccharide chain. However, since commercially obtainable QDs are typically functionalized with carboxylic acids or amines, a derivatization was required. Derivatization was performed on QD-COOH and involved the use of EDCHCl in the presence of the bis-hydrazide compound, ADH, yielding relatively stable hydrazide-functionalized QDs (QD-hydrazide). The drawback is that ADH, being is homobifunctional crosslinker, can introduce undesirable side reactions. As both functional groups on the crosslinker are identical, they each have the potential of reacting with the same QD, resulting in a closed ring structure that can essentially inactivate that particular region of the QD. However, it is suspected that the spacer arm of the crosslinker lacks the length required to form such a ring structure. Another more likely scenario involves the cross-reaction between a derivatized QD (QD-hydrazide) with an underivatized QD (QD-COOH). This uncontrolled reaction can lead to the undesirable formation of a QD-QD polymer (Figure 8a), but is believed to be minimized when using ADH in excessive quantities during the derivatization.

**Figure 9:**
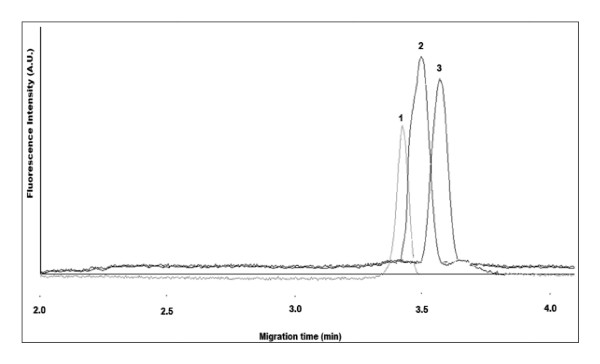
**Overlapping electropherograms illustrating QD-NH_2 _(1), QD-hydrazide (2), and QD-COOH (3)**. CE buffer electrolyte used was 50 mM borate, pH 9.2. Gravity injection performed by elevating inlet capillary 7 cm for 5 s. Applied voltage for CE separation was 28 kV. Capillary temperature maintained at 20°C. Excitation source and detection wavelength was 473 nm and 620 nm, respectively.

Figure 9 illustrates overlapping electropherograms of QD-hydrazide (2) in comparison to QD-NH_2 _(1) and QD-COOH (3). Their characteristic migration times can be attributed to the pKa of the functional group expressed on the QD relative to the pH of the CE separation buffer (9.2). Alkylated primary amines and carboxylic acids have measured pKa ~10, and ~4.5, respectively. Thus, the effect of the CE separation buffer pH allows the QD-NH_2 _to exhibit a net positive charge due to protonation of the primary amines. However, the QD-COOH will be completely ionized, exhibiting a net negative charge. Figure 8 can show a distinct change in migration time between QD-NH_2 _and QD-COOH. Hydrazides have remarkably low pKa values (~2.5), thus QD-hydrazides will be deprotonated during CE separation and exhibit a net-neutral charge. Again, this can be observed in figure 8 as QD-hydrazide migrates intermediate of the positively- and negatively- charged QDs. The small differences in migration times between QDs with substantially different charged residues on their surfaces can be attributed to the very large size of the particles that greatly influence their migration. Suppression of the EOF may improve resolution by means of increased electrophoretic contributions from QD-biomolecule conjugates [33].

The use of QD-hydrazide (in contrast to QD-NH_2_) for bioconjugation with an oxidized IgG (IgG-CHO) increases the selectivity of the reaction. However, there still remains the potential to form undesirable conjugates. The is due to not only the QD-hydrazide containing many reactive sites, but also the IgG-CHO which can contain many polysaccharide chains which can again contain many reactive aldehydes. This can lead to the uncontrolled formation of -QD-IgG-QD- polymers (Figure 8b). However, this undesirable polymer formation can possibly be minimized with using QD-hydrazide in very limited quantities with respect to the IgG-CHO during bioconjugation. Reduced reaction times, temperature, and mildly acidic pH conditions may also prevent undesirable conjugates.

**Figure 10:**
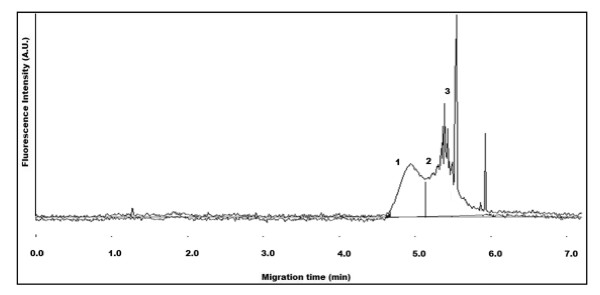
**Non-resolved electropherogram of mixture QD-hydrazide (1) and whole antibody-conjugated QDs (QD-IgG) (2)**. Sharp peaks (3) observed at the migration time of QD-IgG are attributed to buffer additive (BSA) cross-reacting with the antigen-binding site of the IgG. IgG used for conjugation was rabbit anti-human albumin. CE buffer electrolyte used was 50 mM borate (pH 9.2), 0.1% BSA. Gravity injection performed by elevating inlet capillary 7 cm for 5 s. Applied voltage for CE separation was 25 kV. Capillary temperature maintained at 20°C. Excitation source and detection wavelength was 473 nm and 620 nm, respectively.

Figure 10 illustrates the CE separation of QD-hydrazide (1) and their conjugation to whole anti-human albumin IgG-CHO (QD-IgG) (2). The separation is not baseline resolved but can be distinguished by the vertical line separating the two peaks. The QD-IgG, not purified by size-exclusion or dialysis, retains a considerable amount of unconjugated IgG in the sample. This resulted in significant changes in EOF, peak shape, and resolution due to protein-capillary wall adsorption. In addition, the lack of baseline separation could be attributed to the whole IgG exerting a reduced negative charge influence when conjugated to QD-hydrazide. To reduce the effects of protein-capillary wall interaction, a 0.1% BSA additive was included in the CE separation buffer. However, due to the similarities between BSA and the IgG immunogen, human serum albumin (HSA), cross-reactivity may have occurred. The cross-reactivity, leading to a non-specific immunoconjugate (QD-IgG-BSA) may be observed in the electropherogram as sharp spikes, unresolved from the QD-IgG peak.

### Characterization of immunoconjugates

**Figure 11:**
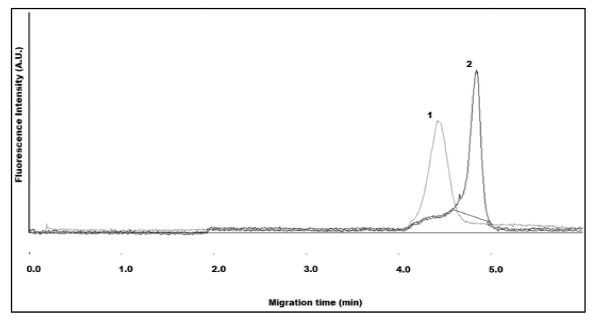
**Overlapping electropherograms illustrating QD-rIgG (1) and antibody's respected immunogen QD-rIgG-HSA (2)**. IgG used for conjugation was rabbit anti-human albumin. Immunogen used was human serum albumin (HSA). CE buffer electrolyte used was 50 mM borate, pH 9.2. Gravity injection performed by elevating inlet capillary 7 cm for 5 s. Applied voltage for CE separation was 25 kV. Capillary temperature maintained at 20°C. Excitation source and detection wavelength was 473 nm and 620 nm, respectively.

**Figure 12:**
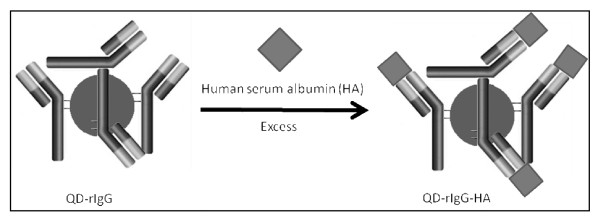
Immunochemical reaction between QD-rIgG and corresponding antigen human serum albumin (HSA).

Figure 11 illustrates overlapping electropherograms of the conjugate QD-rIgG (1) in comparison when exposed to an excess of immunogen, HSA specific for the antibody (see Figure 12 for reaction scheme). There is a significant change in migration time between the bioconjugate QD-rIgG and the resulting immunoconjugate QD-rIgG-HSA (2). The peak fronting observed for the QD-rIgG-HSA overlaps with QD-rIgG and could possibly be due to an incomplete immunochemical reaction. Although the reaction was allowed to take place in the presence of excess HSA, an incubation period of 15 minutes at room temperature may not have been sufficient. The difference in migration time between QD-rIgG and QD-rIgG-HSA was ~25 s. Minimal changes in migration time between successive runs were calculated (~1.8 s) and were attributed to the excess HA present in the sample. However, these changes in migration time due to protein-capillary wall adsorption were not significant in obscuring the detection of an immunoconjugate peak.

## Conclusion

In this paper we used CE-LIF to investigate the bioconjugation of QDs to proteins and immunoglobulins. The electropherograms shown above demonstrate each QD-biomolecule conjugate's electrophoretic behavior. The electropherograms for the various QD-protein, QD-rIgG, and QD-IgG conjugates displayed migration times relatively longer in contrast to QDs prior to conjugation due to increased net-negative charge influenced by the biomolecule. In addition, increased peak broadening was observed with each of the QD-biomolecule conjugates. QD polydispersity and protein/immunoglobulin properties (ie. size, pI, active functional groups for conjugation) were principal contributors for the QD-biomolecule electrophoretic behavior. Various methods for bioconjugation (selective and non-selective) were performed based on the nature of the biomolecule (ie. functional groups available). These bioconjugation techniques, while extensively used with molecular labels, can also be applied for QD labeling. However, due to QDs exhibiting fundamental differences with molecular labels, complications can arise during bioconjugation that can be detrimental to a CE separation. The large size of a QD as well as its vastly functionalized surface can cause a multitude of biomolecules to conjugate with its surface. In addition, biomolecules, particularly proteins and immunoglobulins may contain many functional groups that can actively participate in the conjugation process, leading to an uncontrolled polymerization. Another underlying matter is the significant electrophoretic contribution that the QD gives to the conjugates, due to its large size. An immunoreaction following QD-rIgG conjugation was performed with the IgG's immunogen. The resulting longer migration time for the immunoconjugate suggests a further increase in the immunoconjugate's net-negative charge, however, the peak width displayed no further broadening. Ultimately, this work will continue to evolve in an effort to perform quantum dot-based immunoassays using capillary electrophoresis as an effective and sensitive separation technique. Such work can be directed in the area of infectious biological materials that are generally present in relatively small low concentrations. This work will contribute to the advancements in applying nanotechnology for molecular diagnosis in medical field.

## Competing interests

The authors declare that they have no competing interests.

## Authors' contributions

MP performed all experiments and data analysis in the laboratory. Both authors designed and coordinated experiments. EPCL provided important advice and financial support. MP wrote manuscript. Both authors read and approved final manuscript.
